# Dual Fractions Proteomic Analysis of Silica Nanoparticle Interactions with Protein Extracts

**DOI:** 10.3390/ma17194909

**Published:** 2024-10-07

**Authors:** Marion Schvartz, Florent Saudrais, Yves Boulard, Jean-Philippe Renault, Céline Henry, Stéphane Chédin, Serge Pin, Jean-Christophe Aude

**Affiliations:** 1 LIONS, NIMBE, CEA, CNRS, Université Paris-Saclay, 91191 Gif-sur-Yvette, France; 2 Institute for Integrative Biology of the Cell (I2BC), CEA, CNRS, Université Paris-Saclay, 91198 Gif-sur-Yvette, France; 3 PAPPSO, Micalis Institute, AgroParisTech, INRAE, Université Paris-Saclay, 78350 Jouy-en-Josas, France

**Keywords:** silica nanoparticles, protein extracts, proteomics, mass spectrometry, protein–nanoparticle interactions, corona

## Abstract

Dual-fraction proteomics reveals a novel class of proteins impacted by nanoparticle exposure. Background: Nanoparticles (NPs) interact with cellular proteomes, altering biological processes. Understanding these interactions requires comprehensive analyses beyond solely characterizing the NP corona. Methods: We utilized a dual-fraction mass spectrometry (MS) approach to analyze both NP-bound and unbound proteins in *Saccharomyces cerevisiae* sp. protein extracts exposed to silica nanoparticles (SiNPs). We identified unique protein signatures for each fraction and quantified protein abundance changes using spectral counts. Results: Strong correlations were observed between protein profiles in each fraction and non-exposed controls, while minimal correlation existed between the fractions themselves. Linear models demonstrated equal contributions from both fractions in predicting control sample abundance. Combining both fractions revealed a larger proteomic response to SiNP exposure compared to single-fraction analysis. We identified 302/56 proteins bound/unbound to SiNPs and an additional 196 “impacted” proteins demonstrably affected by SiNPs. Conclusion: This dual-fraction MS approach provides a more comprehensive understanding of nanoparticle interactions with cellular proteomes. It reveals a novel class of “impacted” proteins, potentially undergoing conformational changes or aggregation due to NP exposure. Further research is needed to elucidate their biological functions and the mechanisms underlying their impact.

## 1. Introduction

Nanomaterials (NMs) have become ubiquitous in manufactured products across a diverse range of industries, including food processing, pharmaceuticals, cosmetics and electronics. Among these NMs, silica-based nanoparticles (SiNPs) exhibit the highest prevalence due to their versatile properties and wide applicability [1]. SiNPs find use in various sectors, such as food packaging and additives, materials production (glass, cement, fiberglass, concrete), optics and fiber technologies, textile manufacturing, agricultural fungicides and biomedical/pharmaceutical applications (biological carriers, excipients). This widespread utilization has sparked growing societal concern regarding potential health and environmental consequences [2,3,4], prompting extensive research into the toxicity of NMs. Investigation at the molecular level reveals crucial insights into SiNP interactions with biomolecules. Analyzing protein–SiNP interactions allows for the elucidation of affected biological mechanisms. For instance, studies have demonstrated that SiNPs can specifically target RNA-binding proteins [5]. Furthermore, nanoscale analysis elucidates the influence of SiNP size on corona formation and protein conformational changes [6,7], highlighting the importance of considering both size and surface properties when assessing potential risks or beneficial effects associated with SiNPs.

Understanding the intricate interactions between proteins and nanoparticles (NPs) is crucial for assessing their potential impact on biological systems and guiding the development of safe and effective nanotechnology applications. Numerous methods have been established to elucidate these interactions, with techniques such as fluorescence flow cytometry offering insights into binding events with purified proteins. However, complex protein samples derived from protein cellular extracts, such as those obtained from *Saccharomyces cerevisiae* sp., pose a greater challenge.

In this study, we employ a dedicated protocol detailed in Mathé C. et al. [8] to identify protein–NP interactions within these complex matrices. Briefly, the protocol involves incubating protein extracts with NPs of interest, followed by centrifugation and drying steps to retrieve aggregates of proteins and NPs (hereafter denote “pellet”). Proteins are subsequently desorbed from these complexes for identification using high-throughput label-free nano-liquid chromatography coupled with tandem mass spectrometry (nano-LC MS/MS). Bioinformatics analysis and database harvesting enable protein identification based on spectral matching.

Differential analysis, comparing the identified proteins in exposed samples to those in control samples lacking NP exposure [5], allows us to pinpoint significantly adsorbed proteins. Notably, most existing studies focusing on NP toxicity primarily analyze the pellet fraction, representing the proteins physically bound to NPs. However, this approach overlooks potential impacts on unbound proteins that may undergo structural alterations, aggregation, or other functional changes upon NP exposure. Besides, certain NP types, such as carbon-based NPs, exhibit exceptionally strong binding affinities for proteins [9], rendering traditional desorption methods ineffective. Similarly, smaller plastic particles can aggregate with proteins in solution, preventing their retrieval through centrifugation. In these instances, the fraction containing unbound proteins (hereafter denoted “supernatant”) becomes crucial for a comprehensive understanding of NP-induced effects [10]. It is noteworthy that in most large-scale protein NM interaction studies, usually only one fraction, the pellet or the supernatant, is analyzed.

To our knowledge, no previous studies have implemented a dual analysis approach encompassing both pellet and supernatant fractions within the context of protein–NP interactions. This study aims to shed light on this novel approach by addressing key questions regarding the correlation and complementarity of these fractions. We hypothesize that analyzing both fractions provides a more complete picture of NP-induced protein alterations, encompassing both direct binding events and indirect effects on the proteome. High-throughput mass spectrometry is a sampling procedure, and the number of distinct proteins mapped in a solution is correlated with the proteins’ extract concentration, dynamic and complexity and the acquisition duration. Thus, the proteins identified in the dual fractions are representative samples of the respective population of proteins bounded or not bound to the NP. This sampling effect raises many points of interrogation. In particular, how do these fractions correlate and complement each other? Does the combined pellet and supernatant fractions are representative of the control population (i.e., not exposed to the NP)? What can we learn about the composition of the pellet (*resp.* supernatant) by analyzing the dual supernatant (*resp.* pellet) fraction?

To investigate this hypothesis, we exposed *Saccharomyces cerevisiae* sp. protein extracts to SiNPs. We identified the adsorbed proteins (pellet) and unbound proteins (supernatant) using nano-LC MS/MS, with the spectral count (SC) serving as a proxy for relative protein concentration. Furthermore, we included control samples devoid of NP exposure for differential analysis. In this research, we present a comparison between biological protein concentrations and their corresponding SC ratios obtained from mass spectrometry. Subsequently, we employ various linear models to demonstrate the complementarity of pellet and supernatant fractions. Finally, we utilize differential analysis to highlight the benefits of our dual approach in identifying novel protein subsets impacted by SiNP exposure.

## 2. Materials and Methods

### 2.1. Sample Preparation

**Figure 1:**
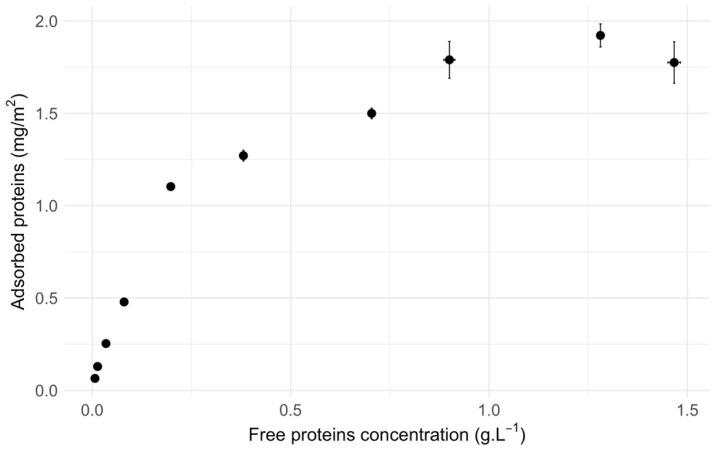
Adsorption isotherm of YPE on silica NP (1 g.L^−1^) in phosphate buffer (100 mM, pH7) conducted by depletion. Ten samples at YPE concentration from 2.5 × 10^−2^ to 2 g.L^−1^ were incubated with silica NP using a ThermoMixer^®^ at 20 °C for 3 h (cycles of 15 s at 800 rpm followed by 285 s at rest). Samples were centrifuged at 20 °C, 20,000 rpm for 10 min, and the supernatant concentration (unbound proteins) was determined using the absorbance at 205 nm with an absorption coefficient of 31 L.g^−1^.cm^−1^. Horizontal and vertical error bars represent the standard error of the mean.

Yeast protein extracts (YPE) were prepared from the *Saccharomyces cerevisiae sp.* strain S288C (Matα SUC2 mal mel gal2 CUP1), as described in [10]. Cell debris was removed during the YPE preparation (cycles of 5 min at 4000 rpm, followed by 40 min at 14,000 rpm). Silica NPs used were a radius 13 nm monodisperse LUDOX TM-50 nanospheres (Sigma-Aldrich, St Louis, MO, USA). Cryo-TEM micrographs of silica NPs with adsorbed oxyHb were given in Sanchez-Guzman D. et al. [11]. LUDOX TM-50 NPs have a limited aggregation in solution [12]. Silica NPs were dialyzed using a membrane with a 3.5 kDa cut-off against Milli-Q water at 4 °C. YPE (0.6 g.L^−1^) was incubated with silica NP (1 g.L^−1^) in phosphate buffer (100 mM, pH7) using a ThermoMixer^®^ (Eppendorf, Hamburg, Germany) at 20 °C for 3 h (cycles of 15 sec at 800 rpm followed by 285 sec at rest). The YPE concentration was chosen at the start of the adsorption isotherm plateau (see Figure 1) to have the minimum protein quantity in saturation conditions.

A centrifugation (20 °C, 20,000 rpm, 10 min) allowed for the separation of free proteins in the supernatant and adsorbed proteins in the pellet. An amount of 500 µL of supernatant was recovered for the proteomic analysis. Then, for the pellet, two washings were conducted as follows: the pellet (40 µL) was resuspended in 1.5 mL of phosphate buffer, centrifuged (20 °C, 20,000 rpm, 5 min) and the supernatant (1.46 mL) was removed. After the two washings, a desorption protocol was performed: the 40 µL of pellet was resuspended in phosphate buffer and sodium dodecyl sulfate (SDS UltraPure^TM^ 10%, Invitrogen, Waltham, MA, USA). The 750 µL final solution contained 1% of SDS, a concentration often used to desorb proteins [6]. Then, mixing for 1 h at 20 °C was conducted using a ThermoMixer^®^ (cycles of 15 s at 800 rpm followed by 105 s at rest). Final centrifugation (20 °C, 20,000 rpm, 10 min) was performed, and the 100 µL of the pellet was recovered for the proteomic analysis. The protein concentration was determined using the peptide bond absorbance at 205 nm with an absorption coefficient of 31 L.g^−1^.cm^−1^ [13].

**Figure 2:**
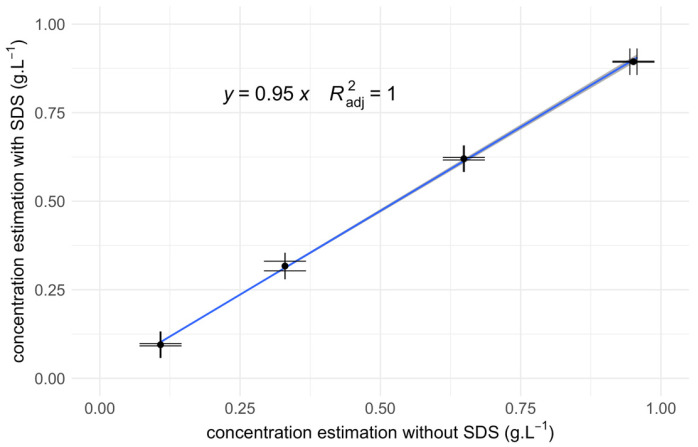
Calibration curve for the YPE concentration with SDS 1%. Concentration levels are determined using the absorbance at 205 nm with an absorption coefficient of 31 L.g^−1^.cm^−1^. The blue curve depicts the linear regression model fitted to the data points. Horizontal and vertical error bars represent the standard error of the mean.

Since SDS impacts the determination of protein concentration, a calibration curve was created to correct the raw concentration values (see Figure 2).

### 2.2. Proteomics Analysis

Tubes containing 60% of their maximum volume of YPE in a phosphate buffer (100 mM) were mixed for 24 h at 3 rpm, 6 °C. Sample protein concentrations were not equalized and were within the recommended range for optimal detection and quantification. Proteomic experiments were performed at the Proteomic Analysis Platform of Paris Sud-Ouest (PAPPSO). A constant volume of YPE samples was deposited on SDS-PAGE gels, and proteins were separated using short migration times. A classic protein digestion protocol was applied (described in Henry C. et al. [14]). Samples were analyzed by LC–MS/MS on an Orbitrap Fusion Lumos Tibrid (Thermo Fisher Scientific, MA, USA) mass spectrometer. The protein identification was performed using the *Saccharomyces cerevisiae* sp. strain S288c protein database (41,6750 entries, v2020).

### 2.3. Spectral Count Normalisation

Spectral counts (SC) were calculated for each protein detected by MS. Then, these raw SC values are normalized in two steps. Firstly, for each fraction, we adjust the SC of the three replicates to a constant average ratio of 40 SC per mg.L^−1^. Secondly, we perform a global normalization to equal the total SC of the pellet and supernatant sum to the control total SC. Normalized SC for each replicate and averaged are shown in Table 1.

Additionally, proteins identified with less than three SC were filtered out from the final datasets.

### 2.4. Statistical Analysis

All statistical analyses were performed using the R language (version 4.2.2) [15], in addition to the standard packages for the correlation analysis, the statistical tests (Pearson and Spearman) and the fitting of linear models. Linear mixed-effects models were adjusted using the “lme4” R package [16]. Generalized linear models used for the Bayesian protein differential analysis (detailed in Marichal L. et al. [6]) were calculated using the following packages: “rstanarm” [17] to fit generalized linear models using a Gaussian link function; “bridgesampling” [18] to compute the log marginal likelihoods; the “BayesFactor” [19] to calculate the Bayes Factors and posterior probabilities from marginal likelihoods.

## 3. Results

### 3.1. Biological Fractions Complementarity

Protein concentration was determined for each technical replicate in all samples using optical density measurements. Concentration measures were adjusted to account for MS sampling bias. Table 2 presents the average protein concentrations for each fraction. As the pellet and supernatant fractions are derived from the control sample, we anticipate their combined protein concentration to closely approximate that of the control. Our data demonstrate a slightly higher total protein concentration for the combined pellet and supernatant fractions (0.73 g.L^−1^) compared to the control (0.63 g.L^−1^). Remarkably, the supernatant and pellet fractions exhibited protein concentrations approximately equal to (50%) or close to (65%) half of that observed in the control sample.

### 3.2. Spectral Counts Distribution Complementarity

Following protein identification via mass spectrometry, the raw spectral count (SC) for each protein was determined as the sum of its corresponding spectra. To account for experimental and technical biases (e.g., sample preparation and mass spectrometry acquisition variability), raw SC values were normalized using a two-step process described in detail within the Methods section. Indeed, the raw SC data exhibited variations in average spectral counts per mg.L^−1^ between fractions: ~40 SC per mg.L^−1^ in the pellet, ~23 SC per mg.L^−1^ in the supernatant and ~20 SC per mg.L^−1^ in the control.

At the protein level, high correlations between technical replicates were observed across all three fractions using both Pearson (range [0.993–1.000]) and the non-parametric Spearman (range [0.902–1.000]) correlation coefficients.

**Figure 3:**
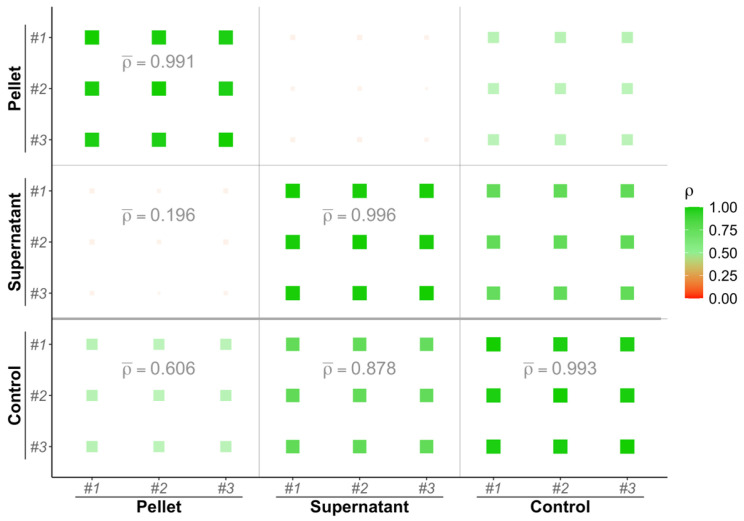
Spectral counts correlation plot between the pellet, supernatant and control fractions. Pearson correlation coefficients between replicates are calculated and depicted as squares wherein the size and color (scale given on the right of the plot) depend on its value. Average correlation coefficient values between fraction pairs are indicated on the lower triangular matrix.

Figure 3 presents a visualization of Pearson correlation coefficient values (represented by a red-to-green color scale). In particular, SC in the control exhibited stronger correlations with the supernatant (ρ = 0.878) compared to the pellet (ρ = 0.606). Conversely, a weak correlation was observed between the pellet and supernatant (ρ = 0.196). These findings underscore the complementary nature of information derived from both the pellet and supernatant fractions relative to the control.

### 3.3. Regression Analysis

To evaluate the complementary nature of the three fractions, control, pellet and supernatant, linear regression models were employed. The independent variable was defined as control spectral counts (SC), while the dependent variables were pellet and supernatant SC. The model equation is represented as:

(1) $$SC_{\mathrm{control}}\;\sim \;\alpha +\beta_{1}\times SC_{\mathrm{pellet}}+\beta_{2}\times SC_{\mathrm{supernantant}},$$

Table 3 presents the fitted coefficients and regression performance metrics. The model demonstrates that both the pellet and supernatant contribute equally to the estimation of control SC, as indicated by the coefficients *β_1_* = 1.07 and *β_2_* = 1.05, respectively. The model exhibits strong adjustment, with an adjusted R^2^ of 0.98 and a Root Mean Square Error (RMSE) of 8.29. Student’s *t*-tests yielded highly significant *p*-values (<0.001) for both the pellet and supernatant coefficients, falling within narrow confidence intervals (see Table 3). The intercept coefficient is nearly zero and was deemed irrelevant to the model based on a Student’s *t*-test *p*-value of 0.83.

To directly compare the “reconstructed” protein subset derived from the combined pellet and supernatant data with the original control set, we summed the averaged spectral counts for each identified protein across the respective fractions. A linear regression model was then fitted to this new dataset, excluding the intercept term (*α* = 0) as previously determined to be irrelevant. The model is defined as:

(2) $$SC_{\mathrm{control}}\;\sim \;\beta \times (SC_{\mathrm{pellet}}+SC_{\mathrm{supernatant}}),$$

**Figure 4:**
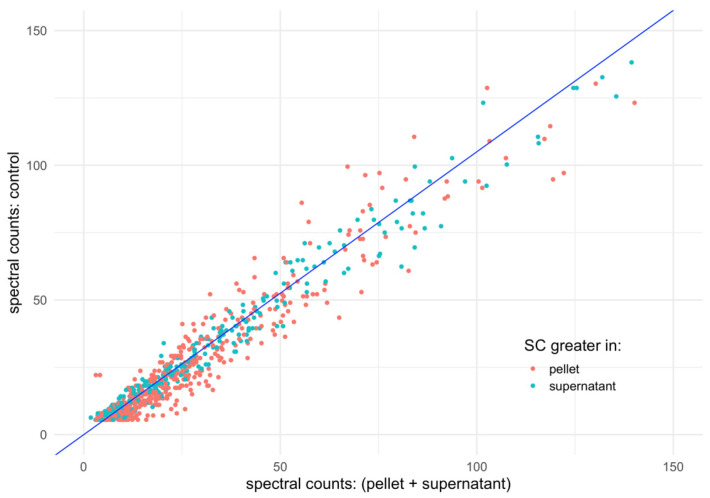
Spectral Counts (SCs) linear regression model. This plot depicts the linear regression as a blue line between the cumulated SC of each protein in the pellet and supernatant, with the SC of these proteins in the control (see Equation (2)). Besides the regression line, each dot depicts the related SC in the control (*y*-axis), cumulated pellet, and supernatant (*x*-axis). The color of the dot is red when the protein is more abundant in the pellet than in the supernatant, and otherwise, it is blue.

The fitted linear coefficient (*β*) for this model is 1.05 ± 0.01, with an adjusted R^2^ of 0.98. Figure 4 visually represents the data, plotting proteins as dots colored according to their majority fraction and overlaying the fitted linear regression line. The strong collinearity between the detected and control sets is evident, with a similar dispersion across each fraction observed for proteins exhibiting the highest SC values.

To assess potential replicate effects on spectral count relationships, linear mixed effects models were employed. The model structure is defined as:

(3) $$SC_{\mathrm{control}}∼\beta_{1}\times SC_{\mathrm{pellet}}+\beta_{2}\times SC_{\mathrm{supernatant}}+(1|replica),$$

where “(1 | *replica*)” in Equation (3) indicates a random intercept for each replicate. Both the pellet and supernatant fitted coefficients remained similar to the initial model, with *β_1_* = 1.07 and *β_2_* = 1.01, respectively. The model exhibited strong adequacy (conditional R^2^ = 0.93 and RMSE = 15.43). Furthermore, an analysis of variance comparing the models with and without replicating mixed effects revealed no significant influence of this parameter (*p*-value < 10^−16^). Based on these findings, we conclude that the random effects attributable to sample replicates are absent.

### 3.4. Differential Analysis between Fractions

**Figure 5:**
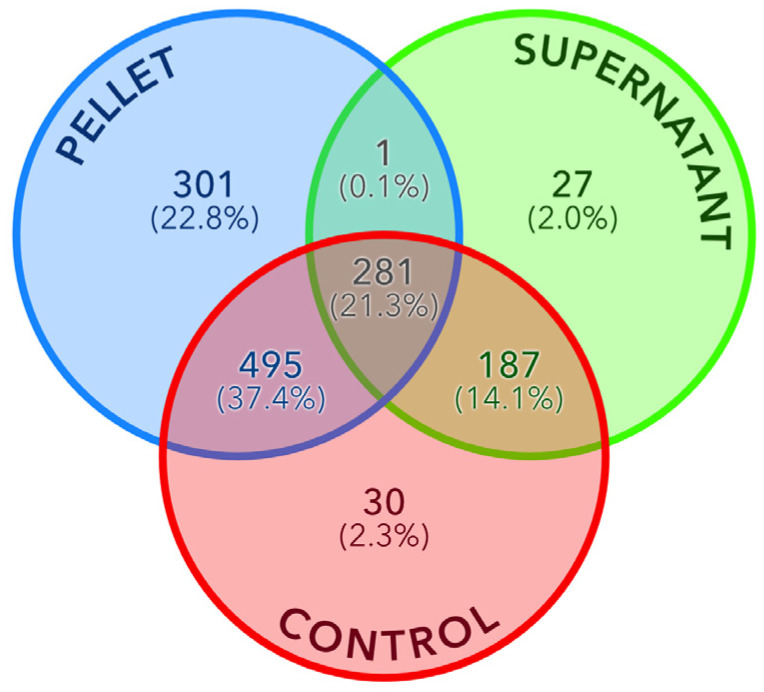
Venn diagram of the number of detected proteins in the pellet, supernatant and control fractions. The relative percentage is indicated in parentheses.

To identify enriched or depleted proteins between the pellet and supernatant fractions relative to the control, a differential analysis was conducted (see Methods section). Prior to this analysis, normalization was performed to equalize the average total spectral counts (SC) across each fraction. Proteins with an average SC below five were subsequently filtered, mitigating potential biases arising from differences in overall protein concentration between samples. Figure 5 presents a Venn diagram illustrating the overlap of proteins identified by MS across the three fractions. Notably, 98% of the proteins identified in the control were also detected in either the supernatant or pellet fraction. The pellet exhibited the highest number of identified proteins (1077), including 301 unique to this fraction.

To determine if differences in spectral counts (SC) between proteins across different conditions are statistically significant, a Bayesian approach was employed (see Methods section). The Bayes factor (BF) served as a threshold for identifying enriched or depleted proteins. This approach effectively distinguished proteins adsorbed onto nanoparticle (NM) surfaces (enriched in the pellet fraction) from those unaffected by the NMs (enriched in the supernatant fraction), as detailed in Table 4.

Table 5 presents a cross-analysis between proteins enriched in the pellet and those depleted in the supernatant for the threshold BF ≥ 3.

Table 5 highlights the distinct protein partitioning patterns based on Bayes Factor (BF) thresholds. At a substantial threshold BF ≥ 3, over 69% (i.e., 124/179) of the pellet-enriched proteins detected in the supernatant are also depleted in this fraction, indicating robust adsorption onto nanoparticles (NPs). Conversely, 59% (i.e., (302–179)/302) of pellet-enriched proteins at this threshold are absent from the supernatant, suggesting highly specific NP binding that results in undetectable levels within the supernatant due to limited sample sensitivity. Notably, no proteins exhibited simultaneous depletion in both fractions (at threshold BF ≥ 3), indicating a low rate of false positives.

The analysis extends to the non-adsorbed proteins present in the supernatant and their relationship with pellet-depleted proteins. Table 6 presents this cross-analysis, demonstrating that at threshold BF ≥ 3, nearly all (over 88.9%) supernatant-enriched proteins detected in the pellet are also depleted in this fraction, reinforcing a clear distinction between adsorbed and non-adsorbed populations. However, a significant disparity exists: 56 non-adsorbed proteins were detected in the supernatant at BF ≥ 3 compared to 302 adsorbed proteins in the pellet. This observation underscores the substantial difference in abundance between these protein groups.

To encompass the full spectrum of protein responses to nanoparticle exposure, we introduce a novel category, “impacted proteins,” encompassing those significantly altered in abundance compared to the control. This category extends beyond simple adsorption/non-adsorption distinctions. We define a protein P as “directly impacted” if it meets the following criteria: (i) P is detected in either the pellet or supernatant fraction, and (ii) P is enriched in the pellet and detected but not depleted in the supernatant. Conversely, a protein P is stated to be “indirectly impacted” if it fulfills criteria (i) and (iii) P is depleted in the supernatant but not enriched in the pellet. The less specific set of “impacted” proteins is the union of the “directly impacted” and “indirectly impacted” proteins subsets.

Applying a BF ≥ 3 threshold (see Table 5), there are 179 − 124 = 55 directly impacted proteins (condition ii) and 265 − 124 = 141 indirectly impacted proteins (condition iii). This analysis reveals a total of 196 proteins impacted by the SiNPs’ exposure at this threshold. Furthermore, 178 (*resp.* 24) proteins were enriched and detected only in the pellet (*resp.* supernatant). These subsets identify proteins with a “very high” bound/unbound affinity for the SiNP surface. Table 7 summarizes the distribution of proteins across each category.

## 4. Discussion

Nanotoxicological investigations strive to elucidate the composition of the nanoparticle (NP) corona, providing crucial insights into the biological mechanisms affected by nanomaterials. Utilizing complex protein extracts enables a broader-scale investigation of molecular mechanisms perturbed by nanomaterials, such as silica nanoparticles (SiNPs), in this study. While conventional molecular biology techniques are effective for analyzing individual or small sets of peptides, deciphering protein–NP interactions within complex matrices necessitates technologies capable of sampling larger sets of proteins. Mass spectrometry (MS) has emerged as one of the preferred methods for high-throughput and large-scale proteomics analyses, proving particularly valuable in characterizing protein–NP interactions [8,18]. 

Most studies employing MS have primarily focused on identifying the subset of proteins shaping the NP corona [20,21,22]. Characterizing the corona offers valuable information for diverse applications; for example, identifying specific proteins within the corona allows for targeted NP functionalization strategies aimed at modulating desired biological interactions. Analyzing the composition of the protein corona can help to decipher the specific molecular pathways impacted by NP exposure, shedding light on the mechanisms underlying potential toxicity or beneficial effects. Identifying the adsorbed proteins on the NP surface requires the following steps: isolation of NP aggregates followed by desorption of bound proteins from the surface and the identification and relative quantification (e.g., spectral counts) using MS. While this protocol effectively identifies adsorbed proteins, it overlooks the unbound proteins remaining in solution, which may exhibit altered fates, such as conformational changes or aggregation, upon NP exposure.

This study demonstrates that a dual fractions approach, encompassing both NP aggregate-bound proteins and unbound proteins in solution, provides a more comprehensive understanding of the proteomic alterations induced by NP exposure in protein extracts. Utilizing SiNPs and *Saccharomyces cerevisiae* sp. protein extracts, we observed strong correlations between protein abundance profiles in both fractions with non-exposed controls (Pearson correlation coefficient *ρ* ranging from 0.6 to 0.9). Notably, minimal correlation was observed between the two fractions themselves (*ρ* = 0.196), indicating distinct proteomic signatures for bound and unbound proteins. Linear models fitting both fractions demonstrated equal contributions to predicting protein abundance in the non-exposed control sample (linear coefficients ranging from 1.05 to 1.07, adjusted R^2^ = 0.98). This suggests that each fraction independently captures unique aspects of the proteomic response to SiNP exposure.

Due to the inherent limitations of MS sampling, which can only detect a subset of the total expressed proteome, a dual-fraction approach provides a more comprehensive understanding of SiNP-induced effects than a single-fraction analysis (e.g., solely focusing on NP aggregates). This study allows us to quantify the sensitivity gain achieved by analyzing a single fraction (the pellet) compared to the dual-fraction approach presented herein. Analysis of the dual fractions resulted in a 22.6% increase in protein detection via mass spectrometry (MS) (single: 1078 proteins detected vs. dual: 1322). Differential analysis of the single fraction to characterize the corona identified 302 proteins whose abundance was significantly higher (threshold BF ≥ 3) compared to an unexposed sample (see Table 4). The dual-fraction approach revealed that 124 of these enriched proteins were detected and depleted in the supernatant fraction. This information allows the distinction between two affinity levels: “very high” for proteins exclusively enriched in the pellet and “high” when they are also detected and depleted in the complementary fraction (see Table 7). Furthermore, these findings also demonstrate that in conditions where proteins cannot be desorbed from NP (e.g., carbon nanotubes [9]) or extracted in solution (e.g., plastic particles), analysis of the subset of depleted proteins in solution is relevant to study the effects of exposure to these particular NPs [10]. Finally, compared to a single fraction analysis, the dual fractions method presented in this article increases the sensitivity of protein detection and assigns affinity levels to the proteins shaping the SiNPs’ corona.

Our results also highlight the utility of the opposite fraction (unbound to SiNPs) for identifying a novel protein subset, those unbound but potentially impacted by SiNPs. Indeed, proteins enriched in one fraction are expected to be depleted in the other. Differential analysis between the supernatant (i.e., proteins in solution) and control fractions at a substantial Bayesian factor evidence level (BF ≥ 3) identified 265 depleted proteins (see Supplementary Table S1), with only 124 also showing enrichment in the pellet fraction (i.e., NP aggregates). This discrepancy underscores the value of considering both bound and unbound protein populations for a comprehensive understanding of protein–nanoparticle interactions. Based on these observations, we propose a formal definition for the subset of “impacted” proteins in the context of dual-fraction proteomic analysis: a protein P, detected at least in one fraction, enriched in the pellet (*resp.* depleted in the supernatant) and detected but not depleted in the supernatant (*resp.* not detected or enriched in the pellet) is directly (*resp.* indirectly) impacted by the NP. This dual-fraction approach expands upon conventional analyses focusing solely on the pellet fraction, which identified 302 proteins (Bayes Factor ≥ 3) adsorbed onto the SiNP surface. Notably, our dual-fraction analysis further reveals an additional 196 proteins impacted by SiNP exposure and 56 proteins non-adsorbed onto the SiNP surface, highlighting the value of considering both bound and unbound protein populations for a comprehensive understanding of protein–nanoparticle interactions.

To the best of our knowledge, this is the first MS-based proteomic study, relying on a dual fractions approach, to analyze both bound and unbound protein populations from YPE exposed to SiNPs. Defining a novel category of “impacted” proteins based on their differential relative abundance in bound and unbound fractions offers a promising avenue to enrich our understanding of the biochemical and biophysical interactions. The “impacted” proteins subset warrants further investigation to elucidate its characteristics; however, several hypotheses can be proposed: Directly interacting proteins may arise from transient binding events, exemplified by the Vroman effect [23], wherein proteins bind either directly to the NP surface (i.e., hard corona) or indirectly via the protein corona (i.e., soft corona) [8,24]; Indirectly impacted proteins could result from protein denaturation or aggregation, facilitated by interactions with plastic surfaces (e.g., tubes) and mechanical agitation [10]. To elucidate the fate of these impacted proteins, some key questions for further research include: Do they undergo conformational modifications? Are they aggregating within the solution? While our results show that this approach is more sensitive and generates a more in-depth analysis of NP exposure, it does require a doubling of the quantities of biological material, sample preparation and MS acquisition times and most probably total costs.

To enhance comparability between samples within our dual-fraction mass spectrometry approach, two key modifications were implemented in the MS analysis protocol: (i) Maintaining non-normalized and unequal sample concentrations was critical. This allowed for a direct relationship between spectral count (SC) values and protein abundance within each sample; (ii) Identical acquisition times were enforced for all samples, ensuring that each sample underwent the same level of sampling depth. These modifications aimed to achieve comparable SC per unit concentration across all samples. However, when sample concentrations fall below detection limits, a correction factor can be applied, followed by normalization, as exemplified with SiNPs (detailed in the Methods section). This approach introduces potential sampling biases. Oversampling may inflate the SC for identified proteins while overlooking low-abundance proteins. Conversely, under-sampling risks suppressing true protein identifications. The implementation of semi-absolute quantification MS techniques [25], employing external standards such as UPS2, could potentially mitigate these biases and optimize the quantitation process. Besides, the robustness of the dual fractions approach could be assessed using matrix validation by comparing several protein extracts with different NPs, similar to the method developed by Blume J.E. et al. [20].

This article demonstrates that a dual-fraction mass spectrometry approach facilitates a more comprehensive understanding of nanoparticle exposure at the molecular level. We introduce a novel protein subset characterized by the absence of corona binding but demonstrable NP impact. In *Saccharomyces cerevisiae* sp. protein cellular extracts exposed to SiNPs, 196 proteins exhibit this characteristic. While a detailed investigation into the biological mechanisms and altered metabolic pathways associated with these impacted proteins is beyond the scope of this work, such an analysis would significantly enhance our understanding of SiNP exposure effects. Future research should explore the applicability of this method to other proteomes, particularly those characterized by larger size or greater protein translation dynamic range [26]. Additionally, optimization of the MS protocol may mitigate current sampling limitations and further improve method efficiency.

## Figures and Tables

**Table 1:** Normalized spectral counts summary. Fractions are indicated in the first column. Columns 2 to 4 contain the total spectral count for each replica. Column 5 indicates the average total SC. Column 6 shows the percentage of the average SC value for each fraction compared to the control average SC.

Fraction	Replica 1	Replica 2	Replica 3	Average	% of Control
Control	20,346	33,223	33,791	29,120 ± 7604	100.0%
Pellet	15,629	17,503	16,068	16,400 ± 980	56.3%
Supernatant	12,453	9768	15,939	12,720 ± 3094	43.7%

**Table 2:** Average protein concentration in each fraction.

Fraction	Average Proteins Concentration
Pellet	0.408 ± 0.005 g.L^−1^
Supernatant	0.318 ± 0.003 g.L^−1^
Control	0.629 ± 0.004 g.L^−1^

**Table 3:** Coefficients of the linear model, Equation (1). For each parameter of the model (first column), the coefficient value and its deviation are indicated in the second column. The third column contains the confidence interval. The Student’s *t*-test statistic and the associated *p*-values are provided in the last two columns.

Parameter	Coefficient	Confidence Interval	*t*-Student	*p*-Value
Intercept (*α*)	−0.09 ± 0.40	[−0.88, 0.70]	29,120	0.83
SC Pellet (*β_1_*)	1.07 ± 0.01	[1.04, 1.09]	16,400	<0.001
SC Supernatant (*β_2_*)	1.05 ± 0.01	[1.03, 1.06]	12,720	<0.001

**Table 4:** Pellet and supernatant Bayesian differential analysis. For Bayesian factor thresholds 3, 10 and 30, we indicate the number of proteins with a significant difference in spectral counts between fractions: pellet and control (columns 2 and 3) and supernatant and control (columns 4 and 5).

Bayes Factor (BF) *Evidence*	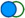 Pellet Fraction		Supernatant Fraction 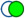	
	Enriched	Depleted	Enriched	Depleted
BF ≥ 3: *substantial*	302	83	56	265
BF ≥ 10: *strong*	139	55	11	159
BF ≥ 30: *very strong*	54	27	2	88

**Table 5:** Cross-analysis of adsorbed proteins in the pellet and supernatant fractions. This table displays the overlap between proteins enriched in the pellet (indicating nanoparticle adsorption) and those depleted in the supernatant (poorly detected in solution) for a Bayes Factor (BF) threshold 3 (see additional Supplementary Table S1 for thresholds 3, 10 and 30). The first column indicates the number of supernatant-depleted proteins. The *blue number* along the top row represents the number of pellet-enriched proteins, and the *green number* indicates the subset of pellet-enriched proteins also detected in the supernatant fraction. The *black numbers* represent the number of proteins concurrently enriched in the pellet and depleted in the supernatant. The *blue (resp. green) percentage* represents the ratio of overlapped proteins to total pellet-enriched proteins (*resp.* and detected in the supernatant).

Depleted in the Supernatant	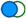 Enriched in the Pellet & Detected in the Supernatant 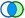			
	302		179	
265	124	41.1%	124	69.3%

**Table 6:** Cross-analysis of non-adsorbed proteins in the pellet and supernatant fractions. This table displays the overlap between proteins enriched in the supernatant (indicating unbound proteins) and those depleted in the pellet (poorly adsorbed) for a Bayes Factor (BF) threshold of 3 (see additional Supplementary Table S2 for thresholds 3, 10 and 30). The first column indicates the number of pellet-depleted proteins. The *green number* along the top row represents the number of supernatant-enriched proteins, and the *blue number* indicates the subset of supernatant-enriched proteins also detected in the pellet fraction. The *black numbers* represent the number of proteins concurrently enriched in the supernatant and depleted in the pellet. The *green (resp. blue) percentage* represents the ratio of overlapped proteins to total supernatant-enriched proteins (*resp.* and detected in the pellet).

Depleted in the Pellet	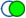 Enriched in the Supernatant & Detected in the Pellet 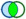			
	56		36	
**83**	32	57.1%	32	88.9%

**Table 7:** Proteins were categorized based on their interaction with silica nanoparticles (SiNPs) using a Bayes Factor threshold ≥ 3. Proteins are classified as: *Unbound* (blue background), proteins not associated with SiNPs; *Impacted* (grey background), proteins showing altered abundance despite not being directly bound to SiNPs; *Bound* (red background), proteins directly interacting with the SiNP surface. Within each category, proteins are further subcategorized: *Very high*, exclusively detected in either the pellet (bound) or supernatant (unbound) fraction; *High*, detected in both the pellet and supernatant fraction; *Direct*, enriched in the pellet and detected but not depleted in the supernatant fraction; *Indirect*, depleted in the supernatant but not enriched in the pellet fraction. Protein counts and relative percentages (%) are indicated in parentheses for each category and subcategory.

Unbound		Impacted		Bound	
**Very High**	High	Indirect	Direct	High	Very High
24 (4.3%)	32 (5.8%)	141 (25.5%)	55 (9.9%)	124 (22.4%)	178 (32.1%)
56 (10.1%)		196 (35.4%)		302 (54.5%)	

## Data Availability

The original contributions presented in the study are included in the article/Supplementary Materials, further inquiries can be directed to the corresponding authors.

## Supplementary Materials

The following supporting information can be downloaded at: https://www.mdpi.com/article/10.3390/ma17194909/s1, Table S1: Cross-analysis of adsorbed proteins in the pellet and supernatant fractions; Table S2: Cross-analysis of non-adsorbed proteins in the pellet and supernatant fractions. Supplementary File S1 ‘Yeast_SiO2_raw.xlsx’: Excel file containing raw spectral counts for the three replicates of the pellet, supernatant and control; Supplementary File S2 ‘Yeast_SiO2_raw.RData’: R datafile containing a tibble data frame (tidyr R package) with raw spectral counts for the three replicates of the pellet, supernatant and control.
