# Supplementary material for: Dual Fractions Proteomic Analysis of Silica Nanoparticle Interactions with Protein Extracts
Source: Materials (Basel). 2024 Oct 7;17(19):4909. doi: 10.3390/ma17194909 (PMC11478063; doi:10.3390/ma17194909)
Supplement: Supplementary file 1 [file materials-17-04909-s001.zip › materials-3209758-supplementary/Supplementary Material_Rev1.pdf]

Supplementary Material

Table S1: Cross analysis of adsorbed proteins in the pellet and supernatant fractions

This table displays the overlap between proteins enriched in the pellet (indicating nanoparticle adsorption) and those depleted in the supernatant (poorly detected in solution) for three Bayes Factor (BF) thresholds (3, 10, and 30). The first column indicates the number of supernatant-depleted proteins based on each BF threshold. *Blue entries* along the top row represent the number of pellet-enriched proteins at each BF threshold. *Green entries* within the blue row indicate the subset of pellet-enriched proteins also detected in the supernatant fraction. *Black entries* in the central section represent the number of proteins concurrently enriched in the pellet and depleted in the supernatant. *Blue percentages* within each column represent the ratio of overlapped proteins to total pellet-enriched proteins at a given BF threshold. *Green percentages*, calculated relative to the subset of pellet-enriched proteins also detected in the supernatant, reflect the proportion enriched in both fractions. For example, with a BF ≥ 10, there are 32 proteins significantly enriched in the pellet and depleted in the supernatant, representing 23% of all pellet-enriched proteins at this threshold (*i.e.* 32/139) and 53.3% of those detected in both fractions (*i.e.* 32/60).

		BF ≥ 3 *(substantial)*		BF ≥ 10 *(strong)*		BF ≥ 30 *(very strong)*	
		**Enriched in the pellet** / **AND detected in the supernatant**					
**depleted in the supernatant**		**302** / **179**		**139** / **60**		**54** / **19**	
BF ≥ 3 *(substantial)*	**265**	124	41.1% / 69.3%	44	31.7% / 73.3%	14	25.9% / 73.7%
BF ≥ 10 *(strong)*	**159**	80	26.4% / 44.7%	32	23.0% / 53.3%	12	22.2% / 63.2%
BF ≥ 30 *(very strong)*	**88**	45	14.9% / 25.1%	21	15.1% / 35.0%	9	16.6% / 47.4%

Table S2: Cross Analysis of non-adsorbed proteins in the pellet and supernatant fractions.

This table displays the overlap between proteins enriched in the supernatant (indicating unbound proteins) and those depleted in the pellet (poorly adsorbed) for three Bayes Factor (BF) thresholds (3, 10, and 30). The first column indicates the number of pellet-depleted proteins based on each BF threshold. *Green entries* along the top row represent the number of supernatant-enriched proteins at each BF threshold. *Blue entries* within the blue row indicate the subset of supernatant-enriched proteins also detected in the pellet fraction. *Black entries* in the central section represent the number of proteins concurrently enriched in the supernatant and depleted in the pellet. *Green percentages* within each column represent the ratio of overlapped proteins to total supernatant-enriched proteins at a given BF threshold. *Blue percentages*, calculated relative to the subset of supernatant-enriched proteins also detected in the pellet, reflect the proportion enriched in both fractions.

		BF ≥ 3 *(substantial)*		BF ≥ 10 *(strong)*		BF ≥ 30 *(very strong)*	
		**Enriched in the supernatant / AND detected in the pellet**					
**depleted in the pellet**		**56 / 36**		**11 / 4**		**2 / 1**	
BF ≥ 3 *(substantial)*	**83**	32	57.1% / 88.9%	4	36.4% / 100%	1	50% / 100%
BF ≥ 10 *(strong)*	**55**	28	50.0% / 77.8%	3	27.2% / 75%	1	50% / 100%
BF ≥ 30 *(very strong)*	**27**	17	30.3% / 47.2%	3	27.2% / 75%	1	50% / 100%
